# Experts’ recommendations for the management of adult patients with cardiogenic shock

**DOI:** 10.1016/j.aicoj.2026.100038

**Published:** 2026-03-31

**Authors:** Nadia Aissaoui, Clement Delmas, Hamid Merdji, Guillaume Schurtz, Guillaume Baudry, Antoine Beurton, Florence Boissier, Laurent Bonello, Bernard Cholley, Nicolas Combaret, Alain Combes, Charles-Henri David, Daniel De Backer, Pierre Grégoire Guinot, Olfa Hamzaoui, Brahim Harbaoui, Julien Imbault, Nicolas Nesseler, Antoine Kimmoun, Michel Kindo, Guillaume Lebreton, Guillaume Leurent, Bruno Levy, Stéphane Manzo-Silberman, Anne-Céline Martin, Armand Mekontso-Dessap, Imane Adda, Joy Mootien, Alexandre Ouattara, Matteo Pozzi, Etienne Puymirat, Francois Roubille, Antonin Trimaille, Aurore Ughetto, Eric Van Belle, Eric Bonnefoy, Khaldoun Kuteifan

**Affiliations:** a Intensive Cardiac Care Unit, Cardiology Department, Hopital Européen Georges Pompidou, Assistance Publqiue des Hopitaux de Paris, Université Paris Cité, Paris, France; b Intensive Cardiac Care Unit, Cardiology Department, Toulouse University Hospital, Toulouse University, INSERM, UMR 1297, Institut des Maladies Métaboliques et Cardiovasculaires - I2MC, Toulouse, France; c REICATRA, Institu Saint Jacques, Toulouse, France; d Department of Medical Intensive Care, University Hospital of Strasbourg, Nouvel Hôpital Civil, INSERM (French National Institute of Health and Medical Research), UMR 1260, Regenerative Nanomedicine (RNM), FMTS, Strasbourg, France; e Cardiac Intensive Care Unit, Insitut Coeur Poumon, CHU Lille, Lille, France; f Université de Lorraine, INSERM, Centre d'Investigation Clinique Plurithématique 1433, Inserm U1116, CHRU de Nancy, Nancy, France; g INI-CRCT (Cardiovascular and Renal Clinical Trialists) F-CRIN Network, Nancy, France; h REICATRA, France; i CHU Bordeaux, Department of Cardiovascular Anesthesia and Critical Care, CHU de Bordeaux, F-33000 Bordeaux, France; j Univ. Bordeaux, INSERM, BMC, U1034, F-33600 Pessac, France; k CHU de Poitiers, Médecine Intensive Réanimation, Poitiers, France; l INSERM, Centre d’Investigation Clinique CIC 14-02 IS-ALIVE, Université de Poitiers, F-86000 Poitiers, France; m Unite de Soins Intensifs de Cardiologie, Hopital Nord de Marseille, Aix-Marseille Université, Marseille 13015 France; n Service d’Anesthésie-Réanimation & Médecine Péri-Opératoire, Hôpital Européen Georges Pompidou, APHP, Paris, France; o INSERM UMR-S1140, Université Paris Cité, Paris, France; p Sorbonne Université, INSERM, UMRS_1166-ICAN, Institute of Cardiometabolism and Nutrition, AP-HP, Service de Médecine Intensive-Réanimation, Institut de Cardiologie, Hopital Pitié Salpêtrière, Paris, France; q Nantes Université, CHU Nantes, Chirurgie Thoracique et Cardiovasculaire, l'Institut du Thorax, F-44000 Nantes, France; r Department of Intensive Care, CHIREC Hospitals, Université Libre de Bruxelles, Brussels, Belgium; s Department of Anesthesiology and Intensive Care, Dijon University Hospital, University of Burgundy Europe, LNC UMR1231, F-21000 Dijon, France; t CHU Reims, Unité de Médecine Intensive et Réanimation Polyvalente, Université de Reims Champagne-Ardenne, UR 3801 PPF, Reims, France; u Interventional Cardiology Department, Hôpital Croix-Rousse, and Hôpital Louis Pradel, Hospices Civils de Lyon, Lyon, France; v University of Lyon, CREATIS UMR5220, INSERM U1044, INSA-15 Lyon, France; w Department of Anesthesia and Critical Care, Pontchaillou, University Hospital of Rennes, Univ Rennes, CHU Rennes, Inserm, CIC 1414 (Centre d’Investigation Clinique de Rennes), Inrae, Institut NUMECAN – UMR_A 1341, UMR_S 1241, FHU SUPORT, F-35000 Rennes, France; x Université de Lorraine, CHRU de Nancy, Service de Médecine Intensive et Réanimation Brabois, U1116, F-CRIN INI CRCT, Nancy, France; y Department of Cardiac Surgery, Hôpitaux Universitaires de Strasbourg, Nouvel Hôpital Civil, Strasbourg, France; z Sorbonne Université, AP-HP, Service de Chrirugie Cardiaque, Institut de Cardiologie, Hopital Pitié Salpêtrière, Paris, France; aa Department of Cardiology, Univ Rennes 1, CHU Rennes, Inserm, LTSI - UMR 1099, Rennes, France; ab Institut du Coeur et des Vaisseaux, CHU Nancy-Brabois, Groupe Choc, équipe 2, Inserm U1116. Faculté de Médecine, Nancy, France; ac AP-HP, Cardiology Department, Cardiology Institut, Hôpital Pitié Salpêtrière, Paris, France; ad Sorbonne University, INSERM, UMRS_1166-ICAN; ACTION Study Group, Paris, France; ae Division of Cardiology, Advanced Heart Failure Unit, AP-HP, Hôpital Européen Georges-Pompidou, F-75015, Paris, France; af Université Paris Cité, INSERM, PARCC, F-75015 Paris, France; ag AP-HP, Hôpitaux Universitaires Henri-Mondor, Service de Médecine Intensive Réanimation, Univ Paris Est Créteil, INSERM, Institut Mondor, CARMAS, Créteil, F-94010, France; ah Department of Cardiac Surgery, Louis Pradel Hospital, Lyon, France; ai Department of Research, One Clinic, PointGyn, Paris, France; aj Unité Fonctionnelle de Conseil en Antibiothérapie, CHU Mulhouse, Mulhouse, France; ak 1CHU Bordeaux, Department of Cardiovascular Anesthesia and Critical Care, CHU de Bordeaux, Univ. Bordeaux, INSERM, BMC, U1034, France; al Department of Cardiac Surgery, Louis Pradel Hospital, Hospices Civils de Lyon, Research on Healthcare Performance Reshape, Inserm U1290, Université Claude Bernard Lyon 1, Lyon, France; am Assistance Publique-Hôpitaux de Paris (AP-HP), Hôpital Européen Georges Pompidou, Department of Cardiology, 75015 Paris, France; an PARCC, Université de Paris Cité, 75006 Paris, France; ao PhyMedExp, Cardiology Department, Université de Montpellier, INSERM, CNRS, INI-CRT, CHU de Montpellier, Montpellier, France; ap Department of Cardiovascular Medicine Nouvel Hôpital Civil, 2UR 3074, Translational Cardiovascular Medicine Biomedicine Research Centre of Strasbourg, University of Strasbourg, Strasbourg, France; aq Department of Cardiothoracic Anesthesia and Critical Care Medicine, Montpellier University Hospital, Montpellier, France; ar Department of Cardiology, Louis Pradel Hospital, Hospices Civils de Lyon, University of Lyon, INSA-Lyon, University Claude Bernard Lyon 1, Lyon, France; as Medical Intensive Care Unit, GHRMSA, Mulhouse, France

**Keywords:** Cardiogenic shock, Guidelines, Temporary circulatory support, Heart team

## Abstract

The last specific international European recommendations regarding the management of cardiogenic shock (CS) regardless of the etiology were issued over 10 years ago. We present herein recommendations for the management of CS in adults, developed using the Grading of Recommendations Assessment, Development, and Evaluation (GRADE) system by an expert group of from the French Intensive Care Society [Société de Réanimation de Langue Française (SRLF)] and the French Society of Cardiology [Société Française de Cardiologie (SFC)], with the participation of the French Society of Anesthesia and Intensive Care [Société Française d'Anesthésie et de Réanimation (SFAR)], and the French Society of Thoracic and Cardiovascular Surgery [Société Française de Chirurgie Thoracique et Cardio-Vasculaire (SFCTCV)].

The recommendations covered six fields of application: CS teams and expert centers, symptomatic medical management, etiological management, organ support, temporary circulatory support and de-escalation and early post-CS management. Twenty-three “Patient Intervention Comparator Outcome” (PICO) questions were identified, leading to 41 recommendations regarding management of CS in adult patients. Seven recommendations were scored with high level of evidence (Grade 1), 11 with moderate level of evidence (Grade 2) and 17 with low level of evidence (Expert opinion). In 6 cases, the experts were not able to give an answer. All of the recommendations obtained strong agreement from the expert committee.

The experts highlight the fact that optimal management of CS requires organization including a structured, multidisciplinary shock team and regional referral network, applying standardized protocols for diagnosis and staging. Early etiological treatment—such as culprit-lesion revascularization or urgent valve intervention—is central to improve outcomes. Hemodynamic support should prioritize norepinephrine as first-line vasopressor and privilege selective inotrope use. Temporary mechanical circulatory support (Impella, VA-ECMO) should be reserved for carefully selected patients following discussion by the expert team.

## Introduction

Cardiogenic shock (CS) is a life-threatening acute cardiac failure syndrome leading to persistent hypoperfusion, entailing short-term mortality of 30–40% and exceeding 50% at one year. While acute myocardial infarction remains a major cause, cases related to acute or acute-on-chronic heart failure (HF) have been on the rise, underscoring the heterogeneity of CS [[1], [2], [3], [4]].

Recent advances include refined classifications such as the Society for Cardiovascular Angiography and Interventions (SCAI) staging system, which effectively stratifies prognosis [5] and guides management, and the growing adoption of multidisciplinary “shock teams” and regional networks. Though numerous position papers and reviews are published every year, the most recent specific international guidelines on management of CS regardless of the etiology were issued over 10 years ago [2,6].

Over the past decade, multiple studies have investigated CS and its symptomatic treatments, including inotropes and mechanical circulatory support, but most have yielded negative results [[7], [8], [9], [10]]. In contrast, and despite the increasing performance of percutaneous interventions for severe valvular disease, randomized trials specifically addressing etiological management remain scarce [11].

To address this gap, the French-Language Society of Intensive Care (Société de Réanimation de Langue Française (SRLF)) and the French Cardiology Society (SFC) convened a multidisciplinary task force to develop evidence-based recommendations using the Grading of Recommendation Assessment, Development and Evaluation (GRADE) approach. Twenty-three “Patient Intervention Comparator Outcome” (PICO) questions were identified across six domains of CS care, leading to 41 recommendations regarding management of CS in adult patients. These guidelines aim to provide an updated, pragmatic framework for clinicians managing this complex syndrome.

## Methodology

### Expert panel

These guidelines focused on the management of CS in adult (≥18 years) patients. The guidelines were developed using the GRADE methodology by an expert committee comprising members of the SRLF and the SFC, with the participation of the French Society of Anesthesia and Intensive Care [Société Française d'Anesthésie et de Réanimation (SFAR)], and the French Society of Thoracic and Cardiovascular Surgery [Société Française de Chirurgie Thoracique et Cardio-Vasculaire (SFCTCV)].

The various disciplines contributing to the management of CS in adults were represented as follows: cardiology, intensive care, anesthesia and cardiac surgery.

### Scope and definitions

We included adult patients presenting with CS due to acute myocardial infarction and acute decompensated HF-CS (ischemic and non-ischemic). Neonates and pediatric patients were excluded.

CS patients due to pulmonary embolism (according to the recent European Society of Cardiology [ESC] guidelines) [12], endocarditis (according to the recent ESC guidelines) [13], myocarditis (according the recent ESC guidelines [14], mixed shock post-cardiac arrest, septic cardiomyopathy, shock following cardiac surgery and drug intoxication were excluded.

### Methods

Key questions were identified by the organizing committee. They were formulated in a Patient Intervention Comparator Outcome (PICO) format following an initial meeting of the expert group. The PICO questions defined the scope of the literature search. The task force of experts also defined keywords for literature searches, determined the time frame for these searches, identified the target populations, and specified the specific outcomes to be addressed [15].

The literature was analyzed using the GRADE methodology [16]. Initially, a level of evidence was assigned to each bibliographic reference based on study design and methodological quality. Subsequently, an overall level of evidence was determined among the experts for each PICO question according to the GRADE methodology. The GRADE process distinctly separates evaluation of the quality of evidence from the strength of the recommendation statements. This separation allows for incorporation of the balance of the risks and benefits associated with adoption of the recommendation. Consequently, even with relatively weak evidence, a recommendation can be deemed “strong” if the net benefits outweighed the harms.

A high overall level of evidence (GRADE 1+: should be used, treated, done/ GRADE 1-: should not be) led to a “strong” recommendation. A moderate level of evidence resulted in a “moderate” recommendation (GRADE 2+: should probably/ GRADE 2-: should probably not). When the literature was insufficient or nonexistent, expert opinion was used to formulate recommendations (experts suggest or can be).

The proposed recommendations were presented and discussed at two expert meetings. Each expert rated each recommendation on a scale ranging from 1 (complete disagreement) to 9 (complete agreement). All experts voted on recommendations. The collective rating was established using a GRADE grid methodology. To approve a recommendation, at least 50% of the experts had to agree, and fewer than 20% could disagree. For strong agreement, at least 70% of the experts had to agree. If strong agreement was not achieved, the recommendations were revised and re-rated to reach consensus. The wording of all recommendations strictly followed the predefined GRADE-based methodology. In this framework, the use of terms such as ‘should’ versus ‘should probably’ directly reflects the strength of the recommendation and the level of evidence, with ‘should probably’ corresponding to a moderate recommendation (GRADE 2), derived from a structured expert consensus and formal voting process integrating both evidence quality and degree of agreement [14,15].

### Results

Twenty-three PICO questions were identified, leading to 41 recommendations regarding management of CS in adult patients. Seven recommendations were scored with a high level of evidence (Grade 1), 11 with a moderate level of evidence (Grade 2) and 17 with a low level of evidence (Expert opinion). In 6 cases, the experts were not able to give an answer

All recommendations obtained strong agreement of the expert committee.

**Area 1: Cardiogenic shock team and expert center**

**R1. CS patients should probably be managed by a multidisciplinary CS team.**

*Level of evidence, grade 2+*

**Fig. 1:**
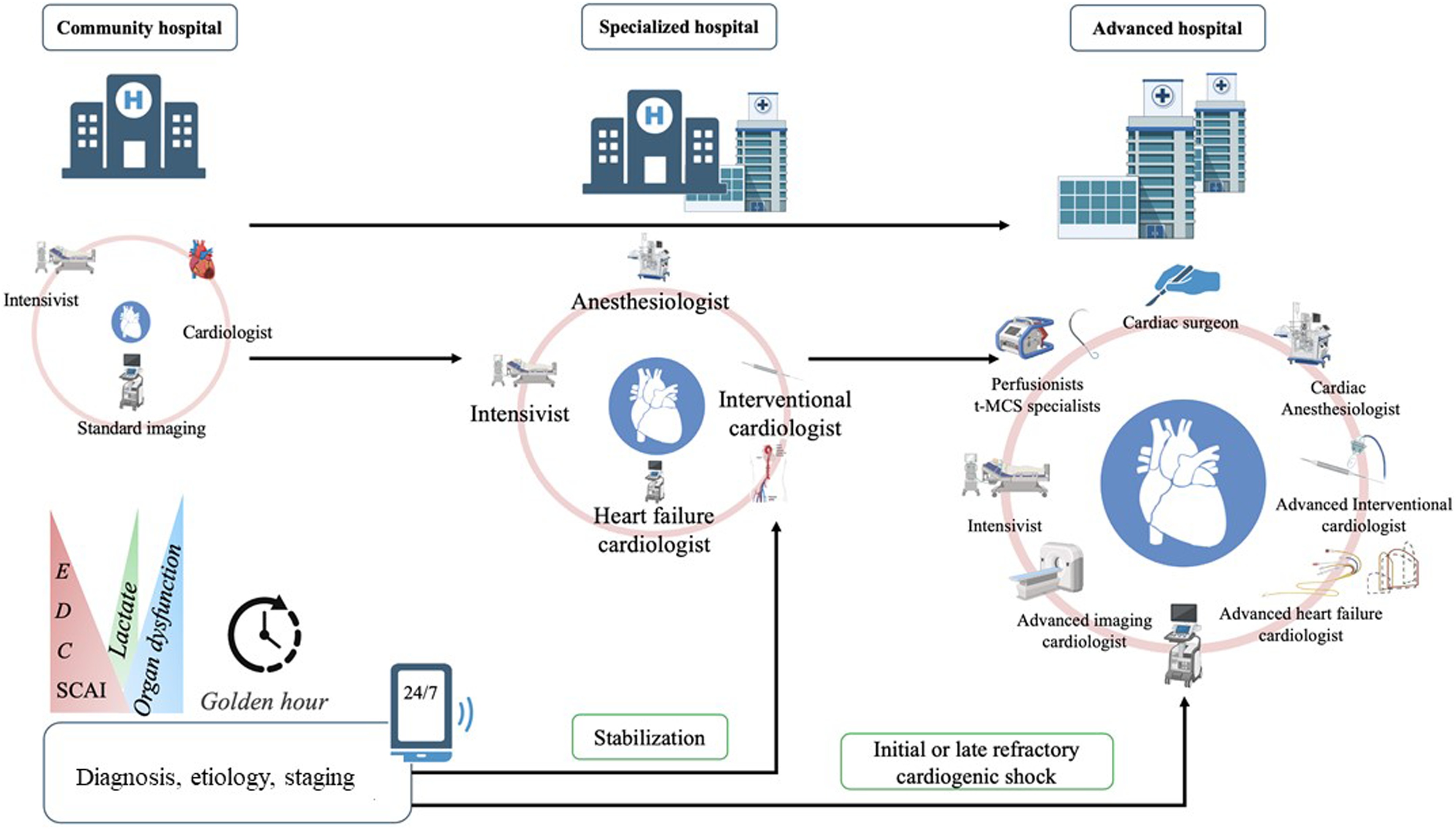
Organization for cardiogenic shock care. CS management is structured across three levels: Community centers ensure initial stabilization and early transfer. Specialized centers manage non-refractory or short-duration shocks (<24 h) with a multidisciplinary team (heart failure specialist, interventional cardiologist, intensivist) focusing on both interventional and medical management. Advanced centers treat refractory or prolonged shocks and candidates for advanced therapies (temporary MCS, VA-ECMO, LVAD, heart transplantation). Refractory CS is defined by persistent hypoperfusion despite optimal therapy. Early recognition and Shock Team activation are key to timely escalation of support. SCAI, Society for Cardiovascular Angiography and Interventions.

**Rationale.** A shock team should be structured based on the available specialties within the institution and the accessibility of key technical platforms, ensuring 24/7 availability (Fig. 1) [2]. The multidisciplinary team should comprise clinicians with expertise in the recognition and diagnosis of CS, accurate assessment of its severity and staging, delivery of advanced critical care, and timely implementation of evidence-based management strategies, including the initiation of temporary mechanical circulatory support (t-MCS) when appropriate [[13], [14], [15], [16]].

Five studies, including a total of 911 patients, reported that implementation of a shock team strategy was associated with a significant increase in in-hospital survival, ranging from 20% to 34%. In-hospital survival rates within shock team-managed groups varied between 54% and 76% [[17], [18], [19], [20], [21], [22], [23]]. The results were unfortunately limited by the study design (single-center studies; applying pre–post intervention methodology). While two additional retrospective studies applying the same methodology did not observe a significant difference in in-hospital mortality, they reported significant improvement in long-term survival in the shock team-managed group [24,25]. Furthermore, a multicenter retrospective registry study including 1242 patients demonstrated that hospitals with an established shock team had significantly higher in-hospital survival rates compared to those without a dedicated team [26]. Morbidity outcomes have been less extensively investigated. Two studies reported that in a shock team strategy was associated with reduced dialysis requirements [24,25].

**R2. Experts suggest discussing and managing patients with CS through a structured regional network centered on specialized shock expert centers with a multidisciplinary shock team, ensuring adequate referral and management of patients based on available resources and expertise.**

*Level of evidence, expert opinion*

**Rationale.** Several expert opinions have proposed different organisational models for CS management, adapted to institutional resources and interprofessional collaboration, yet unified in their objective of optimizing coordination and efficiency so as to improve clinical outcomes [[27], [28], [29]]. To date, no studies have directly compared different shock team compositions.

The implementation of standardized protocols for the diagnosis and classification of CS (e.g., using the SCAI classification [30]), and for monitoring and biomarker-based follow-up, has been shown to minimize delays and inconsistencies in clinical decision-making. Several studies have demonstrated significant improvements in patient outcomes following the adoption of such protocols [18,19,31,32]. Additionally, protocols clearly define the roles and responsibilities of each team member, reducing ambiguity and preventing conflicts related to task allocation [33,34].

**Area 2: Symptomatic medical management**

**R3. In patients with CS, the experts suggest discontinuing chronic heart failure treatments at the time of CS diagnosis so as to avoid worsening of hemodynamic status.**

*Level of evidence, expert opinion*

**Rationale.** Chronic HF medications are well-described in the latest international guidelines [35,36] and include angiotensin-converting enzyme inhibitors, angiotensin II receptor antagonist or angiotensin receptor–neprilysin inhibitors, beta-blockers, mineralocorticoid receptor antagonists, and sodium–glucose cotransporter-2 inhibitors. Even though, when used together, these medications (the “four pillars”), have synergistic effects, as they reduce (all-cause and cardiovascular mortality) and HF hospitalizations, improving HF patients’ quality of life, they also have many side effects, including hypotension, bradycardia and risk of kidney function deterioration.

There has been no randomized trial comparing continuation versus discontinuation of beta-blockers in the time of CS diagnosis. In a post-hoc analysis of the TRIUMPH study, increased mortality was observed in patients receiving beta-blockers within the first 24 h of ischemic CS diagnosis [37]. However, a post-hoc analysis of the multicenter Frenshock registry suggested that in patients with CS of various etiologies, no excess mortality was associated with the continuation of beta-blockers. That much said, the population receiving beta-blockers was significantly less severe than the untreated group [38].

Similar to beta-blockers, no randomized trials have assessed the continuation of ACEi or ARBs in the time of CS diagnosis. In the post-hoc analysis of TRIUMPH, early introduction of cardiovascular treatments (beta-blockers and angiotensin-converting enzyme inhibitors/angiotensin II receptor antagonists) was associated with increased mortality [37]. However, this was significant only in subgroup analyses for the beta-blocker group, and not for those receiving angiotensin-converting enzyme inhibitors or angiotensin II receptor antagonists (but *n* = 11). Given the primarily hypotensive pharmacological effects of angiotensin-converting enzyme inhibitors and angiotensin II receptor antagonists, along with their sometimes-prolonged half-life, prescription of this therapeutic class is not recommended in this setting. Currently, no data exist on the use of sodium–glucose cotransporter-2 inhibitors or mineralocorticoid receptor antagonists in CS. However, the associated metabolic risks, such as euglycemic ketoacidosis, and renal risks, such as acute kidney injury, suggest that these drug classes should not be continued at the time of CS diagnosis.

**R4. Experts suggest not initiating diuretics in patients with CS and significant hemodynamic instability characterized by the need for high-dose vasopressors and/or inadequate cardiac output.**

*Level of evidence, expert opinion*

**Rationale.** A review of the literature found no data specifically addressing the benefits of systematic diuretic administration in CS patients with acute pulmonary edema, as opposed to thise with acute HF [39]. Based on best practice and the SCAI shock classification, diuretics should generally be avoided in patients with hemodynamic instability (SCAI D and E) and considered only in earlier stages (SCAI A, B and for some SCAI C). Evidence from acute HF cohorts suggested a potentially positive impact of diuretics on mortality and rehospitalization rates [[40], [41], [42]]. For specific CS phenotypes with congestion, hypervolemia and/or cardio renal-syndrome, diuretics may be discussed by the CS team, especially for acute decompensated heart failure CS patients (HF-CS). When indicated, loop diuretics are suggested as first-line therapy [43], preferably via the intravenous route, with no clear advantage of bolus versus continuous infusion [44].

***R5. Vasopressors***

**R5A. Norepinephrine should be used as a first-line vasopressor in CS patients.**

*Level of evidence, grade 1+*

**R5B. Dopamine should not be used in CS patients requiring vasopressors unless it is the only vasopressor available.**

*Level of evidence, grade 1-*

**R5C. Epinephrine should probably not be used in CS patients requiring vasopressors.**

Remark: However, experts recommend considering epinephrine if no other options are available or as a last-resort rescue strategy.

*Level of evidence, grade 2-*

**R5D. There is no evidence to recommend the use of vasopressin or angiotensin 2 in CS patients.**

**Rationale.** Norepinephrine acts primarily by increasing peripheral vasoconstriction through α₁-adrenergic stimulation, with a modest β₁-inotropic effect and minimal chronotropic response. As such, norepinephrine should not be considered a pure vasopressor but rather an inopressor, combining potent α₁-mediated vasoconstriction with moderate β₁-adrenergic inotropic activity. This β₁ effect may enhance myocardial contractility and cardiac output in selected patients, particularly in the presence of myocardial depression [45,46]. This pharmacological profile enables effective restoration of mean arterial pressure (MAP) while avoiding excessive tachycardia and a subsequent increase in myocardial oxygen consumption. In the few randomized controlled trials and large-scale observational studies available, norepinephrine has consistently demonstrated a safety profile superior to dopamine in cases of cardiogenic shock. In the SOAP-II trial, which included 280 patients in the cardiogenic shock subgroup, dopamine was associated with higher 28-day mortality and more frequent arrhythmias than norepinephrine [43]. Similarly, in the CardShock registry, epinephrine exposure was independently linked to increased 90-day mortality [47]. In the OptimaCC randomized trial enrolling 57 patients with AMI-related shock after reperfusion, epinephrin led to higher incidence of refractory shock, tachycardia, and lactic acidosis, whereas norepinephrine achieved comparable cardiac output improvements without adverse metabolic effects, even though this safety outcome was defined *a posteriori* by the DSMB [44]. A subsequent individual patient meta-analysis pooling 2583 patients across 16 cohorts confirmed these findings, showing that epinephrin use was associated with an approximately fivefold increase in short-term mortality (adjusted OR ≈ 4.7) [48]. Finally, a pilot RCT in dopamine-resistant cardiogenic shock reported similar increases in cardiac index between norepinephrine + dobutamine and epinephrine; however, epinephrine induced a higher heart rate, more arrhythmias, and metabolic acidosis, thereby reinforcing norepinephrine’s superior tolerability [49]. Taken together, these data position norepinephrine as the vasopressor of choice in cardiogenic shock, offering more stable hemodynamics and less metabolic stress compared with epinephrine [49,50,48,47]. Conversely, epinephrine provides potent β₁ and β₂ stimulation, which can increase cardiac output, but often at the expense of marked tachycardia, elevated myocardial oxygen consumption, and the development of hyperlactatemia due to β2-adrenergic stimulation of glycolysis. These metabolic effects may contribute to the worsening of myocardial ischemia and refractory shock [[44], [45], [46]]. Furthermore, the occurrence of supraventricular or ventricular arrhythmia during epinephrine therapy is frequent and may compromise the already fragile ventricular function in cardiogenic shock. For these reasons, its use should be restricted to specific situations such as cardiac arrest, where its strong vasopressor and inotropic effects can help restore spontaneous circulation, or as a rescue agent when norepinephrine fails to achieve target perfusion pressures.

In mixed or vasoplegic forms of cardiogenic shock, where profound vasodilatation coexists with pump failure, addition to norepinephrine of vasopressin, a pure vasopressor, may represent a useful adjunct. Acting through V₁a receptors, vasopressin restores vascular tone independently of adrenergic pathways and may help reduce catecholamine requirements. However, as with any vasopressor lacking β₁-adrenergic activity, vasopressin increases arterial pressure primarily by raising systemic vascular resistance and therefore left ventricular afterload, which may further impair cardiac output in severe pump failure. At low-dose infusion (0.01–0.04 IU/min), it can reverse refractory hypotension in patients already exposed to high doses of norepinephrine, although excessive vasoconstriction or ischemic complications must constantly be suspected so as to minimize their consequences. Evidence supporting vasopressin in pure cardiogenic shock remains scarce and limited to studies in postcardiac surgery or ECMO populations, which suggest a benefit in catecholamine-sparing strategies [51].

Angiotensin II represents another non-adrenergic alternative targeting the renin–angiotensin system. By acting on AT₁ receptors, it induces potent vasoconstriction and may restore vascular responsiveness in patients with severe vasoplegia or adrenergic receptor down-regulation [49]. As a pure vasoconstrictor without intrinsic inotropic properties, angiotensin II also increases left ventricular afterload, and its use therefore requires particular caution in patients with severe myocardial dysfunction. Data from the ATHOS-3 trial and post-hoc analyses indicate potential hemodynamic benefit in selected cases, but evidence concerning isolated cardiogenic shock is scarce [50]. No patient-centered outcomes have been reported, and no data on patients with CS are available. When used, it should always be initiated under invasive hemodynamic and metabolic monitoring so as to prevent excessive afterload increase, which could further compromise left ventricular output.

***R6. Inotropes***

**R6A. Inotropes should probably be used in CS patients.**

*Level of evidence, grade 2+*

**R6B. Either Dobutamine or Milrinone should probably be used as first-line inotrope in CS patients.**

*Level of evidence, grade 2+*

**R6C. There is insufficient data to favor one inotrope over another as the first-line choice in CS patients, including those receiving beta-blocker therapy.**

**R6D. Epinephrine should probably not be used as inotrope in CS patients.**

Remark: However, experts recommend considering epinephrine if no other options are available or as a last-resort rescue strategy.

*Level of evidence, grade 2-*

**Rationale.** Most of the international guidelines and narrative reviews on the management of CS advocate for use of an inotrope to restore tissue perfusion [4,51,49,50]. However, there has been no trial comparing inotrope versus placebo in patients with CS. The Capital DOREMI2 trial (NCT05267886) a multicenter, double-blind, randomized, placebo-controlled trial, which began recruitment in 2022, is designed to address this gap. This trial attempts to determine whether 12 h of inotrope (dobutamine or milrinone, according to treating physician discretion) achieves better outcomes as compared to 12 h of placebo during the initial phase of CS (SCAI Shock class C or D). After 12 h, physicians are allowed to shift to the open-label drug of their choice.

While the 2016 ESC heart failure guidelines recommended dobutamine as a "first-line" inotrope [48], current guidelines provide no explicit first-line inotrope recommendation [36]. However, dobutamine is recognized as the most widely used inotrope in patients with CS [47,52]. Its widespread adoption is likely attributable to its historical precedence, dating back to the 1970s, and its favorable pharmacokinetic profile, particularly its short half-life, solidifying its position as a common first-line inotrope worldwide [53].

The DOREMI trial [7] is a multicenter, double-blind, randomized controlled trial that enrolled 192 patients with CS (SCAI Shock class B to E) so as to compare the effects of milrinone vs. dobutamine on clinical outcomes. The study found no significant difference between these two drugs in terms of the primary composite outcome, which included in-hospital death from any cause, resuscitated cardiac arrest, receipt of a cardiac transplant or t-MCS, nonfatal myocardial infarction, transient ischemic attack or stroke, or initiation of renal replacement therapy (RRT). Secondary outcomes, encompassing inotrope duration, cardiac ICU length of stay, lactate normalization, and arrhythmia leading to medical team intervention, showed no statistically significant difference. These findings suggest that despite pharmacologic differences, milrinone and dobutamine yield comparable clinical outcomes in CS. However, the extended half-life of milrinone, approximately two hours (further prolonged in patients with acute or chronic kidney injury) [54], contrasts significantly with the very short half-life of dobutamine (two-minutes), thereby facilitating easier titration and management of dobutamine in acute critical situations.

The latest ESC guidelines suggest that due to its mechanism of action independent of beta-adrenergic receptors, type-3-phosphodiesterase inhibitors, such as milrinone, may be preferred over dobutamine in beta-blocker-treated CS [36]. However, in a post-hoc subgroup analysis of the DOREMI trial, focusing on CS patients treated with beta-blockers, no significant difference in the primary composite outcome was observed between dobutamine and milrinone [55].

Thus, in many countries, milrinone is more expensive than dobutamine and may be subject to restricted access or limited supply, whereas dobutamine is widely available, inexpensive, and familiar to most intensive care teams. These factors, together with dobutamine’s short half-life and ease of titration, continue to support its widespread use as a first-line inotrope in routine clinical practice.

SURVIVE [56], a large, multicenter, double-blind, randomized controlled trial, compared dobutamine and levosimendan in patients with acute decompensated HF without CS, and found no significant difference in the primary outcome (all-cause mortality at 180 days). Safety and tolerability analyses revealed that levosimendan administration was associated with a more pronounced initial decline in both systolic and diastolic blood pressure compared to dobutamine. Furthermore, levosimendan induced a greater and more sustained increase in heart rate, persisting through 5 days, and a tendency toward increased incidence of atrial fibrillation within the first 31 days (*p* = 0.05). Similar findings regarding clinical outcomes, safety, and tolerability in patients with acute decompensated HF were also observed in the REVIVE-I and REVIVE-II studies [57].

However, several meta-analyses [58,59] suggest a potential short-term benefit of levosimendan over dobutamine. These analyses report a trend toward improved survival, but the quality of evidence remains very low, due primarily to heterogeneity between studies, small sample sizes, and potential biases in patient selection and study designs.

Given the lack of specific data on patients in CS, extrapolation of the results should be considered with caution. The LevoHeartShock trial (NCT04020263), a prospective, double-blind, multicenter, randomized controlled trial, is designed to address the evidence gap regarding optimal inotropic strategies in severe CS. It compares early levosimendan versus placebo in patients with CS treated with vasopressors and standard inotropes (first-line dobutamine), with a primary composite endpoint associating 30-day mortality, VA-ECMO, and renal replacement therapy.

Of note, the 2018 ESC International Expert Consensus Document on Takotsubo Syndrome suggested levosimendan as a safe and potentially effective alternative to catecholamine inotropes in Takotsubo syndrome complicated by CS [60], despite limited evidence of its benefit [61].

The latest ESC guidelines suggest that levosimendan may be preferred over dobutamine in beta-blocker-treated CS due to its mechanism of action independent of beta-adrenergic receptors [36]. While a post-hoc analysis of the SURVIVE trial, conducted in acute decompensated HF (rather than CS) indicated potential benefits of levosimendan over dobutamine in patients with chronic HF and beta-blocker therapy [62], the applicability of these findings to CS remains uncertain.

Consequently, while levosimendan remains a therapeutic option in specific scenarios of CS, particularly in patients with β-blocker therapy or Takotsubo syndrome, its routine use over dobutamine for CS lacks robust evidence.

Although the authors of the OptimaCC study, a randomized controlled trial comparing epinephrine to norepinephrine-dobutamine in AMI-CS, could not demonstrate a difference in mortality between groups, the study was interrupted early due to an excess of refractory shock in the group receiving epinephrine (37% vs 7%; *p* = 0.008) [63]. Owing to the numerous unwanted side effects of epinephrine in CS [64], most international guidelines currently advocate norepinephrine over epinephrine [65].

**R7. In the absence of evidence, experts make no recommendation regarding the interest of sedation analgesia to decrease myocardial oxygen consumption in CS patients without respiratory or neurological failure.**

**Rationale.** Baseline oxygen consumption (VO2) is reduced by 10 to 15% under the effect of intravenous anesthetic agents [66], mainly due to decreased sympathetic nervous system activity partially attributed to decreased myocardial oxygen demand [67]. Furthermore, a study conducted on a small number of patients demonstrated a decrease in VO2 of approximately 20%, via reduction in the work of breathing, when mechanical ventilation is associated with muscle relaxation in patients with acute HF [68]. However, aside from physiology and pathophysiology studies on small numbers of patients, no clinical study has demonstrated a clear benefit of sedation analgesia in CS. This could be explained by the adverse effects of these molecules; sedation can potentially have adverse hemodynamic effects including decreased cardiac output, systemic vascular resistance, and mean arterial pressure due to reduced sympathetic tone [69].

Occasionally used to relieve dyspnea and anxiety, opioids are nonetheless associated with dose-dependent side effects such as nausea, hypotension, bradycardia and respiratory depression. In acute HF, retrospective studies suggest that morphine administration is associated with a worsened prognosis. More specifically, morphine is associated with respiratory depression, which could lead to mechanical ventilation, prolonged length of hospitalization, increased ICU admissions, and increased mortality [[70], [71], [72], [73]]. Therefore, routine use of opioids in acute HF is not recommended in the latest European guidelines [36]. However, their use may be considered in a targeted manner in patients with severe pain or anxiety, or in a palliative care setting where symptom management is a priority.

**R8. In CS patients, experts suggest considering homologous red blood cell transfusion when hemoglobin level is less than 8 g/dL.**

Remark: A decision to transfuse patients in CS should be driven by non-specific parameters (age, sex, patient history, previous cardiac arrest, hemodynamic and volemia status, bleeding context, …) as is also the case for non-CS patients

*Level of evidence: expert opinion*

**Rationale.** Optimal thresholds for homologous red blood cell transfusion in CS have not yet been scientifically established, and only international consensus statements for unselected critically ill patients are available [74,75].

After adjustment for a propensity score, retrospective North American data on more than 1400 CS patients suggest a favorable association of red blood cell transfusion with hospital survival in patients with hemoglobin < 8 g/dL [76]. This association was not found for hemoglobin ≥ 8 g/dL.

Recent data available in the context of acute coronary syndrome (ACS) provide interesting insights. MINT, an open-label randomized controlled study conducted in more than 3500 patients with ACS, but without details on the number of patients in CS, did not reveal a significant difference in the primary endpoint (ACS recurrence and death at 30 days) between a “restrictive” transfusion strategy with a hemoglobin threshold of 7−8 g/dL and a “liberal” strategy targeting a hemoglobin threshold ≥ 10 g/dL. However, a benefit in terms of reduced cardiovascular mortality was observed as a secondary endpoint. In addition, subgroup analyses revealed a benefit on the primary endpoint in patients with type 1 ACS, chronic or acute HF, impaired left ventricular ejection fraction, or with eGFR < 30 mL/min/1.73 m^2^ [77]. The REALITY trial, a randomized, controlled, open-label study, included over 660 patients with ACS but excluded patients with CS. The primary endpoint at 30 days was a composite of major adverse cardiovascular events (MACE): all-cause death, stroke, recurrent ACS, and emergency revascularization due to ischemia. This trial demonstrated the non-inferiority of a “restrictive” transfusion strategy, recommending transfusion at a hemoglobin threshold <8 g/dL with a hemoglobin goal between 8 and 10 g/dL, compared with a “liberal” strategy recommending transfusion at a hemoglobin threshold <10 g/dL and aiming for a hemoglobin threshold ≥11 g/dL [77,78]. However, given the lack of specific data regarding patients with CS, extrapolation of the results of these two trials to ACS should be approached with caution.

**R9. Therapeutic hypothermia should probably not be used in CS patients.**

*Level of evidence, grade 2-*

**Fig. 2:**
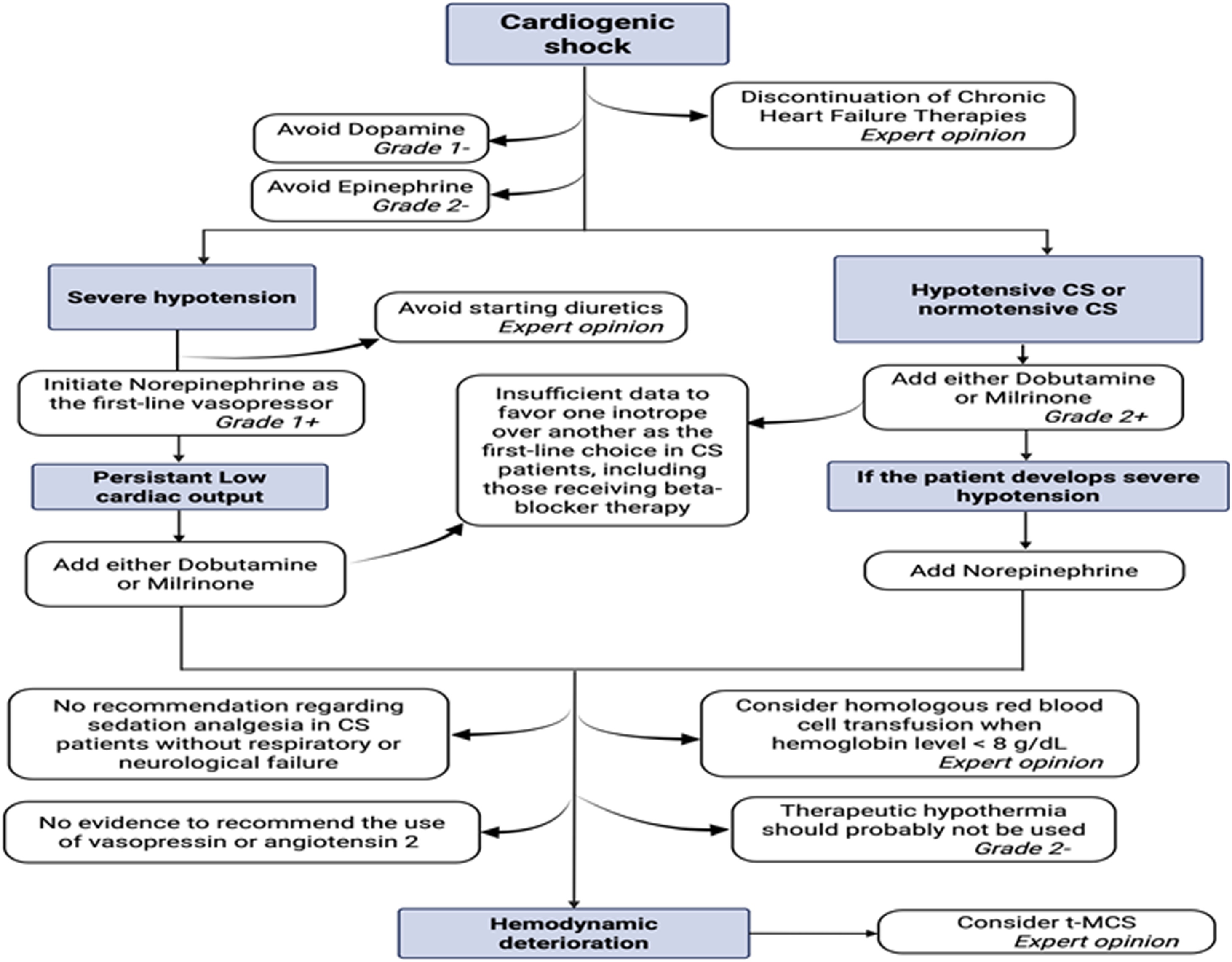
Symptomatic CS management.

***Rationale.*** Intially evaluated in the context of cardiac arrest for its neuroprotective effects, therapeutic hypothermia has been evaluated in the context of CS for its potential effects on hemodynamic parameters, organ dysfunction, and mortality. In a randomized pilot trial involving 20 patients, improved cardiac output and reduced infarct size in patients treated with hypothermia (33 °C) compared with those in normothermia was demonstrated, but without modification of mortality [79]. Nevertheless, in another RCT involving 40 AMI-CS patients, mild therapeutic hypothermia (33 °C) failed to show a substantial effect on cardiac power index at 24 h, and a higher level and slower decline of arterial lactate was found in the interventional arm [80]. Furthermore, therapeutic hypothermia was not associated with improved in mortality. These results were confirmed by the HYPOECMO trial, which showed in a multicenter study on patients with refractory CS supported by VA-ECMO that hypothermia (33−34 °C) did not yield a significant reduction in mortality compared to strict normothermia (36−37 °C) [81]. A meta-analysis corroborating these findings confirmed the absence of a significant effect of hypothermia on mortality, which may nonetheless be associated with improvement in certain hemodynamic parameters [82] (Fig. 2).

**Area 3: Etiological management**

**- Revascularization**

**R10A. In acute myocardial infarction (AMI)-CS patients, coronary angiography should be performed as soon as feasible**

*Level of evidence, grade 1+*

**R10B. In AMI (**ST and non-ST elevation myocardial infarction, **STEMI/NSTEMI) CS patients, revascularization of the culprit lesion by percutaneous coronary intervention (PCI) should be performed as soon as possible so as to improve mid- and long-term survival.**

Remark: Unlike non-CS STEMI/NSTEMI patients, there is no strict time limit for coronary revascularization. But in case of late-presenting AMI-CS (>12 h between chest pain onset and coronary angiography) the decision to reperfuse or not the culprit vessel and the type of procedure to use (PCI vs CABG) may be based on a CS team discussion.

*Level of evidence, grade 1+*

**R10C. In AMI-CS patients with multivessel disease (STEMI/NSTEMI), revascularization by PCI of the infarct-related artery only at the time of the primary angiography, with postponed PCI of non-culprit lesions, should be preferred so as to reduce the composite of early mortality/renal failure.**

Remark: After the initial primary procedure, if the patient remains in shock, staged revascularization should be considered by the CS team balancing the benefit/risk ratio (myocardium at risk, technical aspects of PCI).

*Level of evidence, grade 1+*

**Rationale.** The most important set of information concerning the value of revascularization of the culprit artery in the management of an AMI complicated by CS is derived from the SHOCK trial [[83], [84], [85]]. The SHOCK trial is the only randomized study showing that early revascularization (by PCI, 64% or CABG, 36%) versus medical stabilization, despite having no benefit on 30-day mortality, improved survival at six months and one year. In this trial, the median time between MI and randomization was 12 h, 25% of the population had MI-randomization time >20 h, and no statistical interaction was observed between the MI-randomization time and the benefit of the revascularization strategy. The benefit of an initial revascularisation strategy has been confirmed in many nationwide cohort studies in all patient subgroups, including the elderly [[86], [87], [88], [89]], which underscored the need for an initial invasive management approach in case of AMI-CS.

The CULPRIT-SHOCK study is the only randomized trial focusing on management of the non-culprit arteries during AMI-CS (60% STEMI, 40% NSTEMI). Only at the time of the primary angiography did it demonstrate a significant reduction in all-cause death or RRT at 30-day in the group with PCI of the infarct-related artery [11]. At 1-year follow-up, mortality did not differ significantly between the two groups [11]. Other registry data confirm a trend towards reduced in-hospital mortality with this strategy [90,91]. Of importance, in the “culprit only” group of the CULPRIT-SHOCK trial, a staged revascularization plan was a common and acceptable practice, with 30% of that group undergoing additional PCI within 30 days, and 50–60% within six months.

**R11A. In AMI-CS, experts suggest limiting fibrinolysis to STEMI with no rapid access to coronary revascularization (< 120 min) after initial diagnosis and if the onset of chest pain is < 6 h.**

*Level of evidence: expert opinion*

**Rationale.** No randomized study has been conducted to assess the superiority of systemic thrombolysis over a primary angioplasty strategy in CS patients. Only one large-scale retrospective study has been conducted (5297 STEMI-CS patients treated with thrombolysis compared to 110,452 treated with primary angioplasty), without any difference in terms of all-cause in-hospital mortality even after propensity-matched analysis (30.8% vs. 30.3%, adjusted odds ratio 0.97 [95% confidence interval 0.90–1.05]; *p* = 0.50) [92]. The fibrinolysis group, on the other hand, presented more hemorrhagic complications (13.5% vs. 9.9%; *p* < 0.001) corroborating the results of the older GUSTO-I sub-analyses [93]. Among CS patients, only those treated with PCI showed significantly decreased mortality.

Fibrinolysis essentially lowers the probability of shock occurring in the absence of available primary PCI. This therapy must therefore be available in regions that do not have, or have difficulty in accessing, an interventional technical platform within the timeframes recommended by international recommendations [[94], [95], [96]].

**R11B. In AMI-CS patients, if PCI is not feasible, revascularization of the culprit lesion by CABG should probably be performed as soon as possible to improve mid and long-term survival.**

*Level of evidence, grade 2+*

**Rationale.** In the SHOCK trial, there was no difference in mortality rate with PCI or CABG for patients randomized to early revascularization, with similar survival regardless of the mode of revascularization at 30 days and one year. Additionally, observational studies of patients with CS referred for CABG have reported acceptable outcomes with emergency revascularization [97,98].

**Fig. 3:**
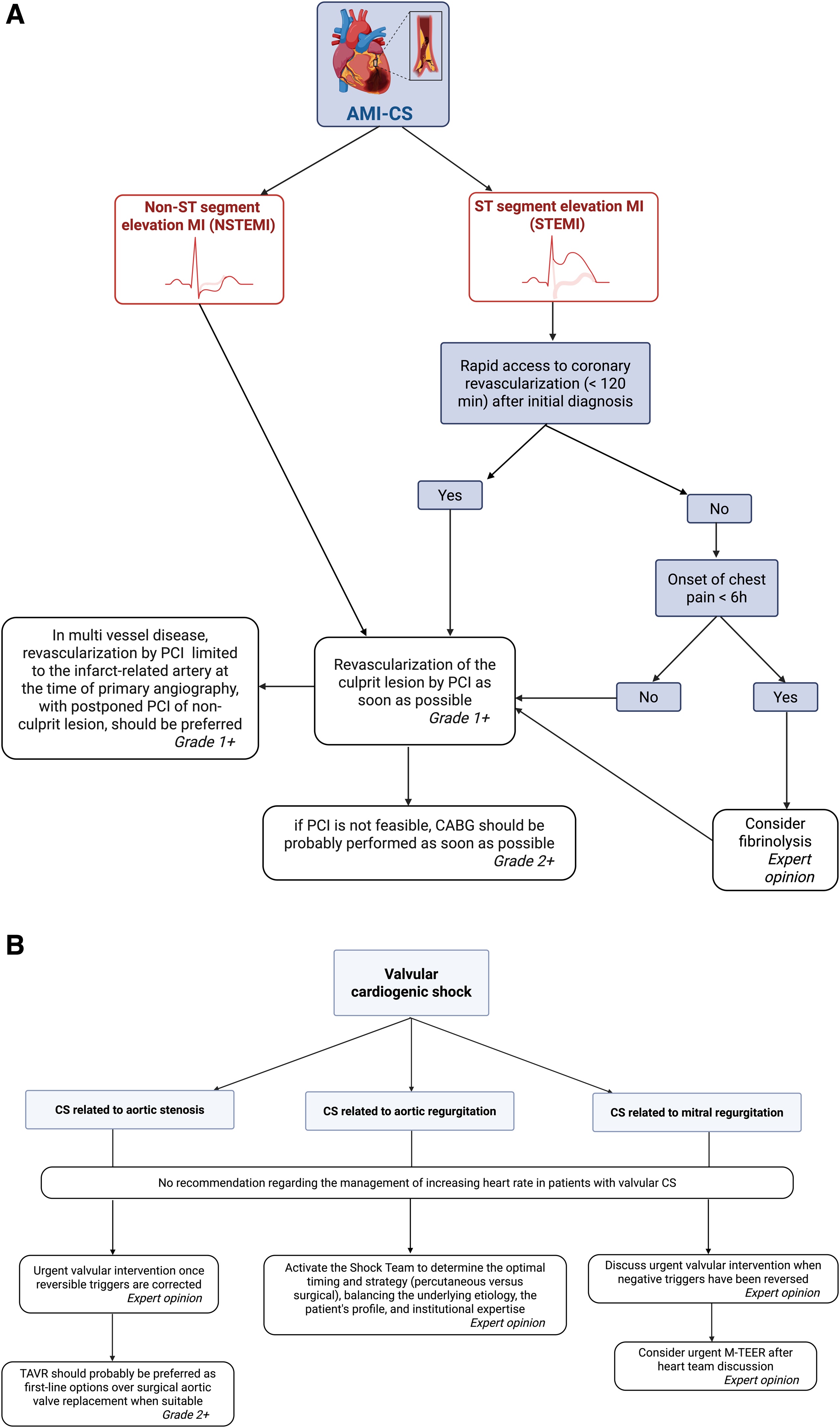
Etiological CS management. A. Etiological management of AMI patients complicating by CS. B. Recommendations regarding other etiological therapies in CS patients.

The recommendations regarding revascularization in AMI patients complicating by CS are summarized in Fig. 3A.

**- Valvulopathy correction**

**R12A. For patients presenting with CS related to aortic stenosis, the expert panel suggests performing urgent valvular intervention when negative triggers have been reversed.**

*Level of evidence: expert opinion*

**R12B. In CS patients, transcatheter aortic valve replacement should probably be preferred over surgical aortic valve replacement as a first-line option, when suitable**

*Level of evidence, grade 2+*

**Rationale.** Aortic stenosis (AS) is the most common valve disease in high-income countries. CS related to AS is associated with very high short-term mortality without intervention [99]. In addition to medical management (trigger correction in cases of myocardial ischemia, tachyarrhythmias, volume shifts) and occasional use of t-MCS, the timing of aortic obstruction relief seems critical [100]. Surgical intervention in critically ill patients is feasible but increases in in-hospital mortality up to 50% [101]. Transcatheter aortic valve replacement (TAVR) has become a class I recommendation (level A) in current guidelines for symptomatic severe aortic stenosis patients ≥ 70 years of age with tricuspid aortic valve [102,103]. CS represents a specific subset of patients excluded from TAVR randomized clinical trials for whom medical treatment alone is an unreliable option, while surgery is often deemed prohibitive. Therefore, guidelines for valvular heart disease management still recommend balloon aortic valvuloplasty (BAV) in CS related to AS for stabilization as a bridge to definitive (surgery or transcatheter) strategy in patients with severe AS requiring urgent high-risk non-cardiac surgery. While BAV may serve as a bridge to definitive therapy, its benefit is transient, and complications such as acute aortic regurgitation are not uncommon [[104], [105], [106], [107], [108], [109], [110]]. TAVR offers better and more sustained hemodynamic improvement potentially translating into better outcomes. This procedure is feasible in acute or emergency settings despite higher complication rates than in elective patients, and with acceptable mortality rate for this very high-risk population [[111], [112], [113], [114], [115], [116], [117]]. Limitations to TAVR expansion in CS remain procedure costs, as well as anatomical or logistical considerations. While optimal definitive aortic replacement strategy in CS remains unknown, a percutaneous approach should always be favored, especially with TAVR [118,119].

**R13. For patients presenting with CS related to aortic regurgitation, the experts suggest activating the CS team to discuss and decide on the timing and the choice of the best strategy to apply (percutaneous versus surgical approach) according to the underlying process, the patient’s profile and local expertise.**

*Level of evidence: expert opinion*

**Rationale.** Aortic regurgitation (AR)–related CS is uncommon but leads to very high in-hospital mortality without timely correction. Etiologies should be clearly dichotomized:

Surgery is generally mandatory in acute AR due to infective endocarditis, acute aortic dissection, or mechanical prosthetic valve dysfunction [13,103].

Percutaneous approaches may be considered when the mechanism is bioprosthetic valve degeneration (Valve-in-Valve TAVR), paravalvular leak, or iatrogenic AR [120].

For native AR in inoperable or extremely high-risk patients, TAVR using new-generation self-expanding or dedicated devices (e.g., JenaValve Trilogy, J-Valve) has recently shown encouraging procedural success (>90%) and (at least) short-term survival in registry data and early feasibility trials. However, TAVR in pure native AR remains technically challenging due to the absence of annular or leaflet calcification, which impairs anchoring and fixation of the transcatheter prosthesis, the frequent presence of large annuli, and the associated risks of device embolization or malposition. These anatomical features require careful preprocedural CT planning and selection of dedicated or oversizing-compatible devices.

While evidence remains limited to small series and registries, procedural refinement and device evolution have extended the percutaneous option to selected cases. The ESC/EACTS 2025 Guidelines now recognize TAVR for pure native AR in inoperable patients (Class IIb, Level of Evidence B) [103]. In the CS setting, decisions must remain individualized and Heart Team–driven, taking into account urgency, reversibility of end-organ failure, and anatomical feasibility [[121], [122], [123]].

**R14A. For patients presenting with CS related to mitral regurgitation, the experts suggest discussing urgent valvular intervention when acute/reversible negative triggers have been reversed.**

Remark: There is insufficient data in the literature to separately consider organic and secondary/functional mitral regurgitation (MR) management in case of associated CS. Nevertheless, in case of severe functional MR (whether de-novo or chronic), heart transplantation or durable MCS should be discussed with the CS team according to patients’ comorbidities and conditions.

*Level of evidence: Expert opinion*

**R14B. For patients presenting with CS related to severe mitral regurgitation, expert suggest considering urgent mitral transcatheter edge-to-edge repair (M-TEER) after heart team discussion.**

*Level of evidence: Expert opinion*

**Rationale.** As MR is the precipitating factor of CS, MR correction seems relevant. Current guidelines recommend surgical management, especially for degenerative (primary) MR. However, surgery in the setting of CS may be challenging, and relevant data are scarce, underscoring the need for a comprehensive multidisciplinary heart team discussion. In 471 post-MI mitral regurgitation patients, including 35% CS, early correction of MR was associated with lower in-hospital and 1-year mortality compared to medical treatment [83].

Data have emerged supporting transcatheter treatment, mainly M-TEER, which is associated with a low complication rate, as a salvage treatment or a bridge to surgery in acute decompensated MR patients [124]. However, the feasibility of M-TEER is sometimes questionable, as anatomical considerations (rupture of the papillary muscle…) may preclude utilization of this technique. A nationwide matched-cohort analysis of 1192 patients hospitalized for CS with mitral valve disease showed significantly lower in-hospital and 1-year mortality in patients treated with M-TEER as compared to those without any MR correction [125]. Moreover, in 3797 patients with CS and significant MR treated with M-TEER, procedural success was associated with lower 1-year mortality or heart failure admissions [126]. The ongoing randomized CAPITAL MINOS trial (NCT 05298124), which aim to evaluate the impact on survival of M-TEER versus standard of care in SCAI C or D CS patients with grade 3+ or more MR, will provide precious data in the future [127].

**R15. In the absence of data in the literature, the panel makes no recommendation regarding increasing the heart rate in CS patients.**

**Rationale.** Increasing the heart rate to improve prognosis in patients with CS (or advanced heart failure) remains debatable, except in the context of shock due to bradycardia caused by complete atrioventricular block or sinus node dysfunction. There are no relevant data available in the literature.

The recommendations regarding other etiological therapies in CS patients are summarized in Fig. 3B.

**Area 4: Organ support**

**Mechanical ventilation**

**R16A. Invasive mechanical ventilation should probably be initiated in patients with CS and acute hypoxemic respiratory failure.**

*Level of evidence, Grade 2+*

**R16B. Given insufficient evidence, the experts cannot make a recommendation regarding non-invasive ventilation as first-line treatment in patients with CS and acute hypoxemic respiratory failure.**

**R16C. If non-invasive ventilation is attempted as first-line treatment, this should be done cautiously by an experienced team, with frequent reassessment and discussion with the intensivist so as not to delay intubation.**

*Level of evidence, expert opinion*

**R16D. Expert suggest not using high-flow nasal canula in patients with CS and acute hypoxemic respiratory failure.**

*Level of evidence, expert opinion*

**Rationale.** Acute respiratory failure is a common and life-threatening complication of CS secondary to increased left ventricular filling pressures and elevated pulmonary capillary pressure. The incidence of respiratory failure varies depending on multiple factors, including stage of CS, underlying pulmonary function, and hemodynamic status of left or right ventricular dysfunction.

Recent observational studies indicate that around one half of the patients admitted to an ICU for CS require ventilation, including invasive mechanical ventilation (IMV), non-invasive ventilation (NIV), and, less commonly, high-flow nasal cannula (HFNC) [[128], [129], [130], [131], [132]].

Positive pressure ventilation can have favorable hemodynamic effects in CS, leading to reduced pulmonary wedge pressure, left ventricular afterload, myocardial oxygen demand, and work of breathing, as well as improved cardiac index and oxygenation [68,133,134].

Two small-scale monocentric studies assessing CS with an intra-aortic balloon pump (IABP) showed that positive end-expiratory pressure (PEEP) could enhance the hemodynamic effects on left ventricular preload and afterload in CS [135,136]. Indeed, PEEP may also enhance myocardial performance and cardiac output in patients with left ventricular failure by reducing excessive cardiac preload while simultaneously lowering left ventricular transmural pressure (i.e., afterload). Conversely, PEEP could impair hemodynamics in case of predominant right ventricular failure. Although ventilation is frequently used, few studies have evaluated its impact on morbidity and mortality. Acute respiratory failure requiring IMV has been associated with higher in-hospital and 30-day mortality in several studies, particularly in patients presenting with severe shock and hypoperfusion signs, and could be a marker of higher illness severity [128,129,132,135]. In post hoc analysis of the TRIUMPH study and CULPRIT-SHOCK study, late intubation was associated with increased mortality [129,135], but precise definition of acute respiratory failure and the criteria for initiation of mechanical ventilation were not described in studies focusing on CS. In the literature of acute hypoxemic respiratory failure without CS, supports other than conventional oxygen therapy have been proposed in case of failure requiring more than 6 L/min of oxygen (i.e. FiO2 at least 40%) or PaO2/FiO2 ≤ 200 mm Hg and a respiratory rate above 25 breaths per minute or clinical signs of respiratory distress [136].

The panel suggests to apply criteria for intubation based on the recent consensus conference on oxygen therapy in acute hypoxemic respiratory failure [137]: cardiac or respiratory arrest, shock requiring vasopressor, persistent or worsening hypoxemia despite maximal oxygenation strategy, recurrent desaturation episodes with SpO2 < 86%, clinical signs of respiratory distress, appearance or worsening of vigilance disorders, respiratory or mixed acidosis, tachypnea with respiratory rate > 30 or worsening respiratory rate, bronchial congestion or copious secretions, agitation, intolerance to oxygenation modality.

IMV should also be initiated when invasive procedures necessitate sedation (e.g., in some cases of revascularisation, t-MCS implantation, or other interventions/surgeries).

Recent observational studies suggest that the positive effects of PEEP may also occur with NIV, which could be used for conscious patients with moderate respiratory failure, without difference on mortality [128,130,138]. However, the level of evidence of these observational studies is very low, and the experts cannot make a recommendation regarding NIV as a first-line treatment.

If NIV is attempted as first-line treatment, this should be done cautiously by an experienced team, following discussion with an intensivist, for selected patients without contraindications such as unresolved hypotension or inability to protect the airways (encephalopathy, coma, vomiting) or to expectorate copious secretions, agitation, uncooperative patient; with evaluation of a correct interface and of patient/ventilator synchrony, and close reevaluation within one hour.

Continuous positive airway pressure or non-invasive inspiratory and expiratory pressure support ventilation could be used, depending on the practice of the centers [139].

Some studies have compared HFNC to NIV in cardiogenic pulmonary edema, but most excluded patients were in shock, and had discordant results. In one RCT and a retrospective study, HFNC appears to have had a higher failure rate due to its less effective application of positive pressure [140,141], whereas other small-scale physiological studies and meta-analyses have suggested that HFNC is not associated with higher risk of treatment failure [[142], [143], [144]].

### Renal replacement therapy

**R17. Experts make no recommendation regarding the early use of renal replacement therapy for patients with CS requiring fluid removal.**

Remark: there is no evidence to consider specific criteria of RRT in CS patients compared to patients with shock due to other etiologies. The same guidelines apply to those patients.

**Rationale.** Fluid removal in patients with CS has the potential to reduce pulmonary and systemic congestion and improve oxygenation. However, evidence directly comparing diuretics to RRT in patients with CS is lacking. The vast majority of the eleven randomized controlled trials evaluating diuretics versus RRT in heart failure excluded patients with blood pressure <90 mmHg, hemodynamic instability, or those receiving inotropes, thereby precluding their inclusion in this analysis [145,146]. Consequently, it is not possible to provide a recommendation regarding RRT versus diuretics for fluid removal in patients with CS. Several randomized trials have evaluated early versus delayed RRT initiation in critically ill patients with severe acute kidney injury without imminent indications for RRT [[147], [148], [149], [150], [151]]. Most studies employed continuous venovenous hemodiafiltration as the RRT technique. Among these, the ELAIN trial [147] enrolled a substantial proportion of patients after cardiac surgery (47%), but none of these trials reported data on the rates of CS, further limiting their applicability in this setting. A single-center pilot RCT by Li et al. [152] explored the feasibility and potential benefits of early RRT initiation within 12 h of ECMO initiation, regardless of conventional RRT indications, in 41 post-cardiotomy CS patients. The study found no significant difference in 30-day all-cause mortality between the early RRT group (61.9%) and the standard care group (75.0%; *p* = 0.51). Lactate clearance was higher in the early RRT arm (0.56 ± 0.4 vs. 0.28 ± 0.4 mmol/L/h; *p* = 0.04). No significant differences in adverse events or hemodynamic instability associated with RRT delivery were observed. In a historically controlled cohort study, Tu et al. [153] evaluated the association of preemptive RRT with survival in 155 post-cardiotomy CS patients. They compared two periods: conventional RRT (initiated based on standard indications) and preemptive RRT (early goal-directed RRT applied to all patients). The study found that hospital mortality was significantly lower in the preemptive RRT group (38.0% vs. 59.2%; *p* < 0.01). Furthermore, the preemptive RRT group presented lower incidence of non-recovery of renal function (4.1% vs. 19.4%; *p* = 0.026) and a shorter time to complete renal recovery (12 ± 15 days vs. 25 ± 15 days; *p* < 0.05). However, the severity of acute kidney injury and hemodynamic failure was greater in the historical cohort, with significantly lower blood pressure, higher lactate levels, and higher vasopressor doses. Given the limited number of studies and their methodological limitations, no recommendations can be made. The two available observational studies are significantly constrained by their design, small sample sizes, and potential biases, preventing any reliable conclusions. High-quality studies are needed to address this critical gap.

**Area 5: Temporary circulatory support**

***Intra-Aortic Balloon Pump (IABP)***

**R18A. The IABP should not be used as a routine temporary mechanical support in CS patients with AMI.**

*Level of evidence, grade 1-*

**Rationale.** Even though intra-Aortic Balloon Pump (IABP) reduces cardiac afterload and myocardial oxygen consumption, while improving coronary blood flow, the increase in cardiac output is limited (0.5 L/minute), compromising its usefulness in CS [154]. Observational data from the fibrinolysis era advocated for wide utilization of the IABP. One small-scale RCT (57 patients with MI complicated by sustained hypotension) found no benefit in 6-month all-cause mortality in the IABP group versus medical treatment only (34 vs 43%, respectively, *p* = 0.23), despite a potential benefit in the subgroup of Killip III and IV patients [155]. Two RCTs have studied the potential benefits of IABP in the contemporary management of MI by primary angioplasty. In a small-scale single-center RCT (*n* = 45), Prondzinski and al. found no improvement in APACHE II score over four days (primary outcome) [156]. The large-scale multicenter RCT IABP-SHOCK II, involving 600 patients, was also neutral for its primary endpoint, all-cause mortality at day 30: 39.7% in the IABP group versus 41.3 % in the control group (*p* = 0.69). All secondary endpoints, *e.g*. in hospital re-infarction or stent thrombosis, serum lactate level, renal function, or length of stay in the ICU, were also similar between groups [157]. Long-term follow-up of this RCT found similar results at one and six years [158,159]. However, the main limitations of the IABP SHOCK II trial should be underlined: it was an open label trial, with a substantial and asymmetrical amount of cross-over (10% of the control group subsequently underwent insertion of an IABP). Moreover, 270 patients (45%) had resuscitation before randomization and 226 patients (37%) were treated by therapeutic hypothermia, in spite of the fact that patients with vasoplegic syndrome might not benefit from IABP. In addition, the authors did not report the cause of death, even though withdrawal of care may have been significant in the event of of cerebral damage. Finally, although the SCAI class was not applied when the study was carried out, enrolled patients were more likely to be in the most advanced class (*i.e.* classes C, D and E). Consequently, the question regarding IABP usefulness remains open in less severe patients (SCAI class B). To sum up, IABP does not improve the prognosis of patients with AMI-CS, and the possible benefits of IABP in less severe patients (SCAI B) remain to be demonstrated.

**R18B. Experts suggest using IABP as the initial temporary mechanical support in CS patients with mechanical complications of AMI as a bridge to surgical or transcatheter repair.**

*Level of evidence, expert opinion*

**Rationale.** Mechanical complications, mainly ventricular septal rupture and MR, nowadays rarely occur following MI. CS in this setting is primarily due to a tremendous decrease of forward stroke volume, combined with an acute rise of pulmonary capillary wedge pressure. Considering its hemodynamic properties, IABP should theoretically be beneficial in management of CS due to mechanical complications [160,161]. However, evidence is scarce and the low incidence of CS due to mechanical complications precludes RCTs. Most of the available evidence comes from case series or registries that describe IABP as a bridge to ventricular septal rupture or MR correction without comparator. In a comparative retrospective study including 46 CS patients with mechanical complications after MI (ventricular septal rupture and MR), Kettner et al. found 100% of 30-day mortality in the eight patients managed without IABP, versus 61% in the 38 patients managed with IABP [162]. In another monocentric retrospective study about 92 CS due to post-MI ventricular septal rupture, including 59 patients on IABP, 30-day all-cause death occurred in 36% in the IABP group vs 94 % in the group without IABP. After adjustment, IABP support was found to be an independent protective predictor of 30-day all-cause mortality (hazard ratio: 0.22; 95% confidence interval: 0.12 to 0.42; *P* < 0.001) [163]. Although, considering its limited hemodynamic support, IABP-related complications are uncommon, escalation to other mechanical supports may be anticipated by the shock team.

**R18C. IABP should not be used as a routine temporary mechanical support in acute decompensated heart failure-CS.**

*Level of evidence, grade 1-*

**Rationale.** IAPB support may be well-suited to the physiopathology of acute decompensated HF-CS, which is characterized by an adaptation to chronic left ventricle dysfunction (chronic low cardiac output) and high resistances with disproportionate increase in afterload, typically the “cold-wet” phenotype [[164], [165], [166]].

In a small-scale RCT encompassing 32 patients with decompensated heart failure and low output, IABP significantly improved organ perfusion at three hours, as assessed by SVO2 (primary endpoint) [167]. Of note, thirty-day mortality was 23% in the IABP group, versus 44% in the standard of care group.

Recently, a multicenter randomized trial (Altshock-2) aimed to assess the effect of early IABP use compared with standard care in 200 CS patients eligible for heart transplantation [10]. The trial was stopped because of futility. Among the 101 included patients, survival at 6o days or successful bridge to heart transplantation was not significantly higher in the IABP group than in the standard care group (81% vs 75%, *p* = 0.45). Complications were comparable between groups. Therefore, routine early IABP use in this patient population did not provide a meaningful benefit in terms of survival or successful bridging to heart replacement therapies.

***Impella***

**R19A. An Impella CP should probably be considered in AMI-CS patients after discussion with CS expert team.**

*Level of evidence, grade 2+*

**Rationale.** The Impella family of devices consists of invasive catheter-mounted left ventricular assist devices that temporarily reduce myocardial workload and oxygen consumption while increasing cardiac output and end-organ perfusion [139]. Three studies have specifically evaluated the outcomes of Impella CP support in AMI-CS, as compared to optimal medical therapy with or without IABP support [[168], [169], [170]]. Among these, only the prospective, multicenter, randomized trial by Møller et al. demonstrated a significant reduction in 180-day mortality associated with Impella CP compared to standard medical therapy in patients with STEMI complicated by CS (hazard ratio (HR), 0.74; 95% confidence interval [CI], 0.55–0.99; *P* = 0.04) [126]. In contrast, the two retrospective propensity score-matched studies found no survival benefit at 30 days with Impella CP support compared to optimal medical therapy alone [169,170]. Regarding safety outcomes, the prospective randomized study by Møller et al. demonstrated a significant increase in adverse events in the Impella CP support group (HR: 4.74; 95% CI: 2.36–9.55). More specifically, there was a significantly higher risk of moderate or severe bleeding (HR: 2.06; 95% CI: 1.15–3.66), limb ischemia (HR: 5.15; 95% CI: 1.11–23.84), and sepsis with positive blood cultures (HR: 2.79; 95% CI: 1.20–6.48) [168]. Similarly, both retrospective studies identified a significantly increased risk of bleeding in the Impella CP group [169,170]. However, none of these studies reported a difference in the incidence of stroke. Given these findings, when considering Impella CP support in AMI-CS, we underscore the importance of a multidisciplinary "shock team" discussion in designated shock centers. This approach is essential to ensuring appropriate patient selection according to the Danger-Shock inclusion criteria and to minimizing the morbidity associated with Impella CP use.

**R19B. The experts suggest considering Impella 5+ (5.0 or 5.5) support for CS patients due to predominant left ventricular failure.**

*Level of evidence: Expert opinion*

**Rationale.** Available evidence on Impella 5+ devices (5.0 or 5.5) is exclusively derived from retrospective, non-controlled studies involving 1804 patients with acute myocardial infarction, acute decompensated heart failure, or postcardiotomy shock; the devices are used as bridge-to-recovery, bridge-to-transplant, or bridge-to-LVAD therapy [[171], [172], [173], [174], [175], [176], [177]]. Reported in-hospital mortality ranged from 6% to 50%, and weaning rates from 42.8% to 93%. In a multicenter registry including 1238 patients, the Impella 5.5 demonstrated higher survival than the 5.0 (70.5% and 88.1%, for AMI and ADHF, respectively) with low complication rates (vascular <0.6%, major bleeding <2.6%, stroke <3.2%, hemolysis <3.2%) [176]. Three North American series (*n* = 921) reported use of Impella 5+ as a bridge to transplantation or LVAD, showing in-hospital mortality of 4.8–24.4%, successful bridging in 70.1–86.2%, and one-year post-transplant survival ≈ 90% [[178], [179], [180]]. Complication rates remained acceptable: stroke 1.8–6.5%, major bleeding 7.7%, limb ischemia 1.8%, and renal failure 8.8–22.2%. Overall, in carefully selected INTERMACS 1–3 patients, Impella 5+ offers effective left-sided circulatory support and serves as a bridge to advanced surgical therapy, with a favorable safety profile compared with VA-ECMO [[180], [181], [182]].

***VA ECMO (Veno-arterial extracorporeal membrane oxygenation)***

**R20A.VA-ECMO should probably not be routinely used in in AMI-CS patients**

Remark: The experts suggest considering VA-ECMO in selected patients with deteriorating AMI-CS after discussion with the shock team.

*Level of evidence, grade 2-*

**Rationale.** VA-ECMO provides full cardiopulmonary support and is particularly suited in case of biventricular dysfunction or after prolonged cardiac arrest [181,182]. By improving myocardial perfusion, reducing ventricular workload, and stabilizing hemodynamics, this device can interrupt the downward spiral of shock and mitigate multiorgan dysfunction. VA-ECMO use has increased substantially with the widespread availability of percutaneous systems and the absence of a proven survival benefit with IABP therapy [183]. If the first observational studies suggested that VA-ECMO could stabilize hemodynamics and improve survival rates in AMI-related CS [[184], [185], [186]], more recent randomized controlled trials have yielded mixed or neutral results, with no significant differences in 30-day mortality or secondary efficacy outcomes, and in some cases with higher incidence of complications, including vascular ischemic events and bleeding. However, these findings should be interpreted with caution due to several biases and limitations in the studies. A major limitation is the large proportion of patients (>70%) with a history of cardiac arrest before VA-ECMO initiation, contributing to significant mortality from neurological injury. Additionally, patient selection was biased, with most participants classified as SCAI stage C, whereas real-world VA-ECMO indications more commonly involve patients in advanced stages (D/E) with severe hemodynamic instability and multi-organ dysfunction, limiting the applicability of the findings to higher-risk populations. Another major limitation is the short VA-ECMO duration observed in these trials, with minimal escalation to heart transplantation or long-term mechanical circulatory support. This contrasts with clinical practice, during which CS patients supported by VA-ECMO are prioritized for advanced therapies based on national heart allocation systems in countries such as France and the US. Furthermore, left ventricular unloading strategies have been underutilized, with only a small proportion of patients (around 5%) in the VA-ECMO group receiving unloading, compared to 30–40% in recent registries. The absence of unloading likely exacerbates the adverse myocardial effects of VA-ECMO, reducing its overall hemodynamic benefit. Finally, the high crossover rates (30%) in the control groups, with emergent use of t-MCS, add complexity to interpretation of the results and may dilute differences between study groups. Consequently, while VA-ECMO may have a role in selected high-risk patients with CS, its routine use should be avoided due to the high complication rates and the limited proven benefits demonstrated in current trials.

**R20B. Experts suggest considering VA-ECMO in selected non-AMI-CS patients after discussion with CS expert team.**

*Level of evidence: expert opinion*

**Rationale.** Few data have described the efficacy and safety of temporary MCS in non-AMI-CS patients and reported encouraging results [187,188]. However, no adequately powered RCT of temporary MCS in this population has been completed [183,189].

In cases of advanced dilated cardiomyopathy with severe CS, VA-ECMO can serve as a bridge to transplantation by stabilizing hemodynamic function and preserving end-organ perfusion [96]. This approach aligns with national heart allocation systems that prioritize these patients for advanced therapies. In such cases, VA-ECMO can be highly effective when used judiciously and in conjunction with a clear transplantation strategy. Overall, experts suggest that the decision to use VA-ECMO in non-ischemic CS should be individualized, taking into account the underlying etiology, the reversibility of the condition, and the patient’s candidacy for advanced therapies.

**R21. There is no evidence to recommend implantation of temporary mechanical circulatory support before rather than after percutaneous coronary intervention in AMI-CS patients.**

**Rationale.** Utilization of t-MCS in the context of AMI-CS is the subject of debate. In the majority of the randomized trials, although there were many differences, the timing of device implantation was not analyzed. In the IABP-SHOCK II trial [157], in which the use of an IABP was not associated with reduced 30-day mortality, the majority of IABP devices were implanted after PCI (37 before and 240 after PCI). There was no between-group difference. In the DanGer SHOCK trial [168] 55% of patients received the Impella CP before PCI, and Impella implantation was associated with lower mortality at 6-month [190]. According to registry data, there was a trend towards better survival when mechanical circulatory support was implanted before PCI [191], particularly when the Impella system was applied through standardized protocol [192].

**Area 6: De-escalation and early post-CS management**

**R22. Experts suggest the initiation of guidelines regarding long-term cardiovascular treatments after the resolution of shock and before hospital discharge.** Remark: Experts suggest discussing therapeutic implementation within the CS team and planning specialized follow-up with a cardiologist

*Level evidence: expert opinion*

**Rationale.** To date, no randomized study has investigated the prescription of cardiovascular treatments in the immediate aftermath of CS to improve prognosis. A systematic review of the literature identified only two retrospective studies evaluating the effects of introducing combined heart failure treatments rather than a single treatment.

In a multicenter retrospective study including 535 patients after hemodynamic stabilization, the combined prescription of beta-blockers, mineralocorticoid receptor antagonists, and either angiotensin-converting enzyme inhibitors or angiotensin II receptor blockers, compared to different regimens (less than drug class 3) was associated with reduced risk of death, with an adjusted one-year hazard ratio of 0.54 (95% CI: 0.29–0.99) [193].

In a second single-center retrospective study including 185 CS survivors with a reduced left ventricular ejection fraction, the authors found no association between the implementation of heart failure treatments and the rate of 30-day readmission. However, patients who were prescribed at least one additional class of cardiovascular treatment had better six-month and one-year survival rates, with adjusted odds ratios of 7 (95% CI: 1.9–28.5) and 6 (95% CI: 1.9–20.5), respectively, compared to those having received no additional treatment [194].

The literature on the management of patients with acute heart failure has consistently reported a benefit from the early initiation of heart failure treatments during hospitalization and the up-titration of doses within six weeks following discharge [195]. Given the side-effects of these treatments—such as arterial hypotension with angiotensin-converting enzyme inhibitors or angiotensin II receptor blockers, negative inotropic and bradycardic effects with beta-blockers, metabolic effects with sodium–glucose cotransporter-2 inhibitors (such as euglycemic ketoacidosis), and renal dysfunction with mineralocorticoid receptor antagonists—these treatments should be initiated only after complete and sustained resolution of the shock state.

**R23. There is no evidence to recommend early mobilization in CS patients.**

Remark: In CS patients stabilized with t-MCS, early mobilization seems to be feasible and safe when supervised by an experienced team.

**Rationale.** Early mobilization and active rehabilitation in mechanically ventilated patients may attenuate ICU-acquired weakness, increase survival without disability and enhance long-term quality of life. Consequently, such interventions have been integrated in international guidelines and of care bundles, but mainly based on observational studies, pilot studies or phase 2 RCTs [196]. However, a recent phase 3 RCT (the TEAM study) found no impact of increased early mobilization on the number of days alive and out of the hospital at 180 days (primary outcome) compared to usual care (including less intensive mobilization) [197]. Secondary outcomes including quality of life, activities of daily living, disability, cognitive function, and psychological function were similar in the two groups. Furthermore, the intervention was associated with increased adverse events. That said, a subsequent large meta-analysis and systematic review, incorporating data from the TEAM study, provided reassurance, reporting an adverse event rate of less than 3% and no overall increase in adverse events or mortality following ICU mobilization [198]. Nonetheless, several key questions remain unresolved, particularly regarding the optimal timing, duration, and frequency of mobilization sessions, as well as the influence of ventilation status, admission diagnosis, and the level of mobilization in the usual care comparator groups. For patients with CS, available evidence is scarce and primarily addresses the safety and feasibility of mobilization in those receiving t-MCS. Two small observational studies (*n* = 24 and 10 patients) demonstrated the feasibility and safety of mobilizing CS patients on IABP awaiting heart transplantation [199,200]. In VA-ECMO patients, larger single-center observational studies (*n* = 35, 63 and 177) and one randomized pilot study (*n* = 7 in the Intervention group) reported similar findings supporting the feasibility and safety of mobilization in this population [[201], [202], [203], [204]].

However, experts found no data on the feasibility or safety in CS patients without t-MCS. Similarly, they found no data on the impact of early mobilization on the short-term or long-term outcome of early mobilization in CS patients with or without t-MCS.

## Sustainability considerations in the management of patients with cardiogenic shock

Intensive care units are major contributors to healthcare’s carbon footprint, with greenhouse gas emissions and waste generation approximately twice that of general wards [205]. Specific treatments required for patients with CS, including revascularization strategies [[206], [207], [208], [209]], valvular interventions [210], electrophysiologic procedures [211], and ventricular assist devices, further amplify the carbon footprint associated with intensive care provision. Future works assessing the environmental footprint of CS management through specific evaluations, including material flow analysis, waste audits, life-cycle assessments and procurement data review, are mandatory to design future sustainable interventions and optimize patient management [212]. When different treatments exhibit a similar level of recommendation, their environmental impact according to the future available evidence may be considered when selecting the most appropriate therapeutic option. Above and beyond the environmental dimension, sustainability considerations are critical in settings with limited resources, such as low- and middle-income countries or during shortages of essential medical devices and drugs, where optimized resource utilization is both an ecological and an ethical imperative. In these contexts, aligning sustainable responsibility with clinical efficiency should be considered as part and parcel of decision-making processes. Beyond those contexts, in order to choose wisely and with proportionality, clinical decision-making always requires evaluation of the patient’s therapeutical perspective by a multidisciplinary team.

## Conclusion and perspectives

CS continues to represent one of the most complex and lethal syndromes in contemporary cardiovascular medicine, with persistently high short- and long-term mortality despite major advances in reperfusion, MCS, and critical care. These joint multidisciplinary guidelines—led by the SRLF, the SFC, and supported by the SFAR and the SFCTCV—provide the first comprehensive, evidence-based recommendations dedicated to the management of adult CS in more than a decade.

Across six major domains encompassing organization of care, symptomatic medical management, etiological treatment, organ support, t-MCS, and post-shock care, forty-one recommendations were established using the GRADE methodology. These recommendations, endorsed by strong expert consensus, reaffirm a central paradigm shift: the management of CS should no longer rely on isolated interventions but rather on coordinated, multidisciplinary, and protocolized strategies.

The implementation of structured “shock teams” and regional referral networks has been identified as a cornerstone of effective CS management. Evidence consistently demonstrates that coordinated multidisciplinary care—integrating cardiology, intensive care, anesthesia, and cardiac surgery—improves survival and reduces morbidity. Standardized protocols for diagnosis, severity staging (e.g., SCAI classification), and escalation or de-escalation of therapy reduce variability, streamline decision-making, and ensure the right intervention at the right time. By the same token, early etiological correction, including culprit-lesion revascularization or urgent valvular intervention, remains the single most powerful determinant of survival.

In terms of hemodynamic stabilization, norepinephrine has been reaffirmed as the first-line vasopressor, while inotropes such as dobutamine or milrinone should be tailored to individual patient profiles. Temporary MCS, including Impella and VA-ECMO, should not be used routinely but rather be reserved for selected patients after multidisciplinary discussion, balancing potential benefit against procedural complexity and complication risks.

Despite progress, the overall level of evidence remains modest, reflecting the scarcity of randomized controlled trials in this heterogeneous population. The expert panel identifies several key research priorities:

• Defining optimal timing, selection, and weaning strategies for t-MCS;
• Refining pharmacologic approaches across distinct CS phenotypes;
• Evaluating early integration of valvular and percutaneous therapies;
• Assessing the long-term impact of standardized multidisciplinary care models on survival and quality of life.

The establishment of national and international prospective registries, harmonized according to standardized classification systems, is an essential next step to generate high-quality evidence, enable benchmarking, and facilitate adaptive, data-driven decision-making.

Finally, the transition from acute stabilization to recovery requires renewed focus. Early reintroduction of disease-modifying heart failure therapies, structured follow-up in specialized centers, and attention to long-term organ recovery and rehabilitation will be key to improving survival beyond the acute phase.

In conclusion, these updated recommendations provide an integrated and pragmatic framework for the management of CS, rooted in multidisciplinary collaboration and evidence-informed practice. Moving forward, the field must evolve from reactive, rescue-based approaches toward standardized, anticipatory, and precision-guided care. Collaborative, multicenter research initiatives and structured regional shock networks will be instrumental in translating these principles into measurable improvements in survival and recovery for patients with CS.

## Funding

CD received consulting and lecture fees from Abbott

DDB received fees for lectures and/or consulting from AOP Pharma, Edwards Lifesciences, Abbott, Pharmazz, Viatris

PGG received fees for lectures from AOP, Medtronic, Edwards and Vygon. PGG is consultant for ABBOT.

EP recieved grants from Abbott, Astra-Zeneca, Bayer and fees and/or consulting from Abbott, Amarin, Amgen, Astra-Zeneca, Bayer, Bouchara-Recordati, Biotronik, BMS, Boehringer Ingelheim, Bracco, Daiichi-Sankyo, Lilly, MSD, Novartis, Novo, Organon, Pfizer, Sanofi, Servier, Sunpharm, Vifor Pharma

BC received research grants from Edwards Life Sciences and Orion Pharma, and consulting/lecturing fees from Edwards, AOP Health, Nordic pharma, and Orion Pharma

The other authors report no conflicts of interest with the contents of this manuscript

## CRediT authorship contribution statement

AISSAOUI reports was provided by Public Assistance Hospitals Paris. AISSAOUI reports a relationship with Public Assistance Hospitals Paris that includes: AISSAOUI has patent pending to NA. Given my previous role as asscoiate editor in AOIC, I had no involvement in the peer review of this article and had no access to information regarding its peer review. Full responsibility for the editorial process for this article was delegated to another journal editor If there are other authors, they declare that they have no known competing financial interests or personal relationships that could have appeared to influence the work reported in this paper.

## Declaration of Generative AI and AI-assisted technologies in the writing process

None.
