# Supplementary material for: Experts’ recommendations for the management of adult patients with cardiogenic shock
Source: Ann Intensive Care. 2026 Mar 31;16:100038. doi: 10.1016/j.aicoj.2026.100038 (PMC13081668; doi:10.1016/j.aicoj.2026.100038)
Supplement: Supplementary file 1 [file mmc1.pdf]

srlf — Société de Réanimation de Langue Française

# Experts' recommendations for the management of adult patients with cardiogenic shock

Société Française de Cardiologie

| Area | | Recommandations | Level of evidence |
|---|---|---|---|
| **Cardiogenic shock teams and expert centers** | | R1. CS patients should probably be managed by a multidisciplinary CS team. | Grade 2+ |
| | | R2. Experts suggest discussing and managing patients with CS through a structured regional network centered on specialized shock expert centers with a multidisciplinary shock team, ensuring adequate referral and management of patients based on available resources and expertise. | Expert opinion |
| **Symptomatic medical management** | **Chronic HF treatments** | R3. In patients with CS, the experts suggest discontinuing chronic heart failure treatments at the time of CS diagnosis to avoid worsening of hemodynamic status. | Expert opinion |
| | **Diuretics** | R4. Experts suggest not to initiate diuretics in patients with CS and significant hemodynamic instability characterized by the need for high-dose vasopressors and/or inadequate cardiac output. | Expert opinion |
| | **Vasopressors** | R5 A. Norepinephrine should be used as first-line vasopressor in CS patients. | Grade 1+ |
| | | R5 B. Dopamine should not be used in CS patients requiring vasopressors unless if it is the only vasopressor available. | Grade 1- |
| | | R5 C. Epinephrine should probably not be used in CS patients requiring vasopressors.<br>Remark: However, experts recommend considering adrenaline if no other options are available or as a last-resort rescue strategy. | Grade 2- |
| | | R5 D. There is no evidence to recommend the use of vasopressin or angiotensin 2 in CS patients. | |
| | **Inotropes** | R6A. Inotropes should probably be used in CS patients. | Grade 2+ |
| | | R6B. Either Dobutamine or Milrinone should probably be used as first-line inotrope in CS patients. | Grade 2+ |
| | | R6C. There is insufficient data to favor one inotrope over another as the first-line choice in CS patients, including those receiving beta-blocker therapy. | |
| | | R6D. Epinephrine should probably not be used as inotrope in CS patients.<br>Remark: However, experts recommend considering epinephrine if no other options are available or as a last-resort rescue strategy. | Grade 2- |
| | **Sedation** | R7. In the absence of evidence, experts make no recommendation regarding the interest of sedation analgesia to decrease myocardial oxygen consumption in CS patients without respiratory or neurological failure. | |
| | **Hemoglobin level** | R8. In CS patients, experts suggest considering homologous red blood cell transfusion when hemoglobin level is less than 8 g/dL. | Expert opinion |
| | **Therapeutic hypothermia** | R9. Therapeutic hypothermia should probably not be used in CS patients. | Grade 2- |
| **Etiological management** | **Revascularization** | R10A. In acute myocardial infarction (AMI)-CS patients, coronary angiography should be performed as soon as feasible | Grade 1+ |
| | 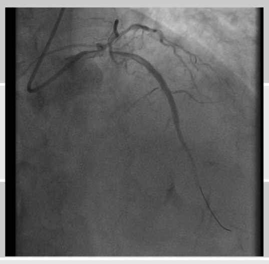 | R10B. In AMI (STEMI/NSTEMI) CS patients, revascularization of the culprit lesion by percutaneous coronary intervention (PCI) should be performed as soon as possible to improve mid and long-term survival.<br>Remark: Unlike non-CS STEMI/NSTEMI patients, there is no strict time limit for coronary revascularization. But in case of late presenting AMI-CS (>12h between chest pain onset and coronary angiography) decision to reperfuse or not the culprit vessel and the type of procedure to use (PCI vs CABG) may be based on a CS team discussion. | Grade 1+ |
| | | R 10C. In AMI-CS patients with multi-vessel disease (STEMI/NSTEMI), revascularization by PCI of the infarct-related artery only at the time of the primary angiography, with postponed PCI of non-culprit lesions, should be preferred to reduce the composite of early mortality/renal failure.<br>Remark: After the initial primary procedure, in case the patient remains in shock, staged revascularization should be considered by the CS team balancing the benefit/risk ratio (myocardium at risk, technical aspects of PCI). | Grade 1+ |
| | | R11 A. In AMI-CS, experts suggest limiting fibrinolysis to STEMI with no rapid access to coronary revascularization (< 120 min) after initial diagnosis and if the onset of chest pain is < 6h. | Expert opinion |
| | | R11B. In AMI-CS patients, revascularization of the culprit lesion by CABG if PCI is not feasible should be probably performed as soon as possible to improve mid and long-term survival. | Grade 2+ |
| | **Valvulopathy correction** | R12. A. For patients presenting with CS related to aortic stenosis, the expert panel suggests performing urgent valvular intervention when negative triggers have been reversed. | Expert opinion |
| | 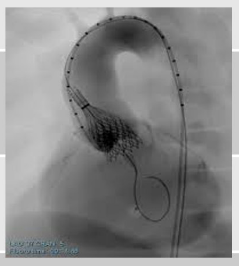 | R12B. In CS patients, transcatheter aortic valve replacement should probably be preferred as first-line option over surgical aortic valve replacement when suitable | Grade 2+ |
| | | R 13. For patients presenting with CS related to aortic regurgitation, the experts suggest activating the CS team to discuss and decide on the timing and the choice of the best strategy to use (percutaneous versus surgical approach) depending on the underlying process, the patient's profile and local expertise. | Expert opinion |
| | | R14A. For patients presenting with CS related to mitral regurgitation, the experts suggest discussing urgent valvular intervention when negative triggers have been reversed.<br>Remark: There is insufficient data in the literature to consider separately organic and secondary/functional mitral regurgitation (MR) management in case of associated CS. | Expert opinion |
| | | R14B. For patients presenting with CS related to severe mitral regurgitation, expert suggest considering urgent mitral transcatheter edge-to-edge repair (M-TEER) after cardiology-based team discussion. | Expert opinion |
| | **Increasing heart rate** | R 15. In the absence of data in the literature, the panel makes no recommendation regarding the increasing heart rate in CS patients. | |
| **Organ's support** | **Mechanical ventilation** | R15A. Invasive mechanical ventilation should probably be initiated in patients with CS and acute hypoxemic respiratory failure. | Grade 2+ |
| | 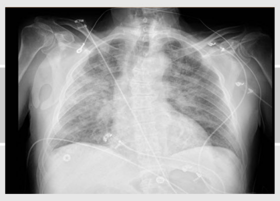 | R15B. Given insufficient evidence, the experts cannot make recommendations regarding non-invasive ventilation as first-line treatment in patients with CS and acute hypoxemic respiratory failure. | |
| | | R15C. If non-invasive ventilation is attempted as first-line treatment, this should be done cautiously by an experienced team with frequent reassessment and discussion with the intensivist so as not to delay intubation. | Expert opinion |
| | | R15D. Expert suggest not to use high flow nasal cannula in patients with CS and acute hypoxemic respiratory failure. | Expert opinion |
| | **Renal replacement therapy** | R16. The experts make no recommendation regarding the early use of renal replacement therapy for patients with CS requiring fluid removal.<br>Remark: there is no evidence to consider specific criteria of RRT in CS patients compared to patients with shock due to other etiologies. The same guidelines apply to those patients | |
| **Temporary circulatory support** | **IABP** 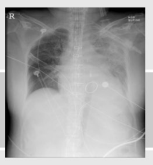 | R17 A. IABP should not be used as a routine temporary mechanical support in CS patients with AMI. | Grade 1- |
| | | R17 B. Experts suggest using IABP as the initial temporary mechanical support in CS patients with mechanical complications of AMI and as a bridge to a surgical or transcatheter repair. | Expert opinion |
| | | R17C. IABP should not be used as a routine temporary mechanical support in acute decompensated heart failure-CS. | Grade 1- |
| | **Impella** 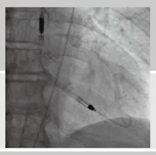 | R18A, An Impella CP should probably be considered in AMI-CS patients after discussion with CS expert team. | Grade 2+ |
| | | R 18B. The experts suggest considering Impella 5+ (5.0 or 5.5) support for CS patients due to predominant left ventricular failure. | Expert opinion |
| | **VA ECMO** | R19A.VA-ECMO should probably not be routinely used in in AMI-CS patients<br>Remark: experts suggest considering VA-ECMO in selected patients with deteriorating AMI-CS after discussion with the shock team. | Grade 2- |
| | 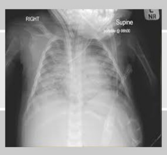 | R19B. Experts suggest considering VA-ECMO in selected non-AMI-CS patients after discussion with a CS expert team. | Expert opinion |
| | | R 20. There is no evidence to recommend implantation of temporary mechanical circulatory support before rather than after percutaneous coronary intervention in AMI-CS patients. | |
| **Desecalation and early post-CS management** | **Chronic HF treatments** | R 21. Experts suggest the initiation of guidelines recommended long-term cardiovascular treatments after the resolution of shock and before hospital discharge.<br>Remark: Expert suggest discussing therapeutic implementation within the CS team and planning specialized follow-up with cardiologist | Expert opinion |
| | | R 22. There is no evidence to recommend early mobilization in CS patients.<br>Remark: In CS patients stabilized with t-MCS, early mobilization seems to be feasible and safe for an experienced team. | |
